# Systemic Sarcoidosis With Cardiac Involvement: Pyrexia With Revived Cardiac Arrest

**DOI:** 10.7759/cureus.90789

**Published:** 2025-08-23

**Authors:** Ashok Kumar, Manish Gaba, Pankhuri Kumari

**Affiliations:** 1 Internal Medicine, Max Smart Super Specialty Hospital, Saket, New Delhi, IND; 2 Microbiology, Amrita Hospital Faridabad, Faridabad, IND

**Keywords:** adult cardiac arrest, cardiac sarcoidosis (cs), general internal medicine, pyrexia of unknown origin (puo), sarcoidosis

## Abstract

A 50-year-old female presented with complaints of intermittent fever during the evening hours for three weeks, associated with decreased appetite and generalized weakness. Clinical examination revealed an erythematous rash over both lower limbs and scleral congestion in the eyes. She was admitted to the general ward for further evaluation. On day 1 of admission, she developed an episode of ventricular fibrillation, which was reverted with cardioversion. She had no past or family history of heart disease. She was intubated during cardiopulmonary resuscitation, placed on mechanical ventilatory support, and subsequently transferred for further management. Post-arrest imaging was performed. Echocardiography showed no regional wall motion abnormality and a normal ejection fraction. Chest radiography revealed bilateral hilar prominence, while CT of the chest demonstrated enlarged lymph nodes in the pre-carinal, pre-tracheal, and subcarinal regions. Blood, urine, and endotracheal secretion cultures were sterile. Laboratory investigations revealed an elevated erythrocyte sedimentation rate, anemia (hemoglobin: 10.8 g/dL), acute kidney injury (serum creatinine: 2.3 mg/dL), and hypercalcemia (12.6 mg/dL). Tracheal secretions were negative for tuberculosis polymerase chain reaction, and no acid-fast bacilli or fungal elements were seen on staining. The angiotensin-converting enzyme (ACE) level was elevated (141.9 U/L). The history of prolonged fever, elevated inflammatory markers, anemia, acute kidney injury, hypercalcemia, bilateral hilar prominence, mediastinal lymphadenopathy, and raised ACE levels prompted consideration of sarcoidosis. Cardiac MRI demonstrated late gadolinium enhancement, suggestive of infiltrative disease. Serum protein electrophoresis was negative for M bands. A skin biopsy taken from the erythematous rash on the lower limb, a cutaneous manifestation of sarcoidosis, revealed non-caseating granulomas. She was diagnosed with systemic sarcoidosis with cardiac involvement. Treatment was initiated with corticosteroids, followed by the addition of a steroid-sparing agent. This case highlights the clinical features, diagnostic workup, and management of a rare presentation of cardiac sarcoidosis.

## Introduction

Cardiac sarcoidosis (CS) is an infiltrative cardiomyopathy resulting from granulomatous inflammation of the myocardium [1]. The annual incidence of systemic sarcoidosis in the United States is estimated at 10.9 per 100,000 in the White population and 35.5 per 100,000 in the African-American population. Cardiac involvement occurs in approximately 5-7% of these patients [1].

Patients with CS may present with symptoms of heart failure (10-30%), conduction abnormalities, or ventricular arrhythmias, which represent a significant cause of sudden cardiac death. Diagnosis remains challenging because of the absence of a single definitive test, the patchy nature of myocardial involvement, and the possibility of isolated cardiac disease in the absence of systemic manifestations.

Advanced imaging modalities such as cardiac MRI and fluorodeoxyglucose positron emission tomography (FDG-PET) play an important role in establishing an early diagnosis. FDG-PET also assists in identifying optimal biopsy sites. Early initiation of corticosteroids and immunosuppressive therapy can improve outcomes and reduce the risk of fatal arrhythmias. Nonetheless, a high degree of clinical suspicion and a multidisciplinary approach are essential for the timely recognition and management of CS.

This case highlights the indolent course of CS and underscores the importance of early diagnosis and appropriate management.

## Case presentation

**Figure 1:**
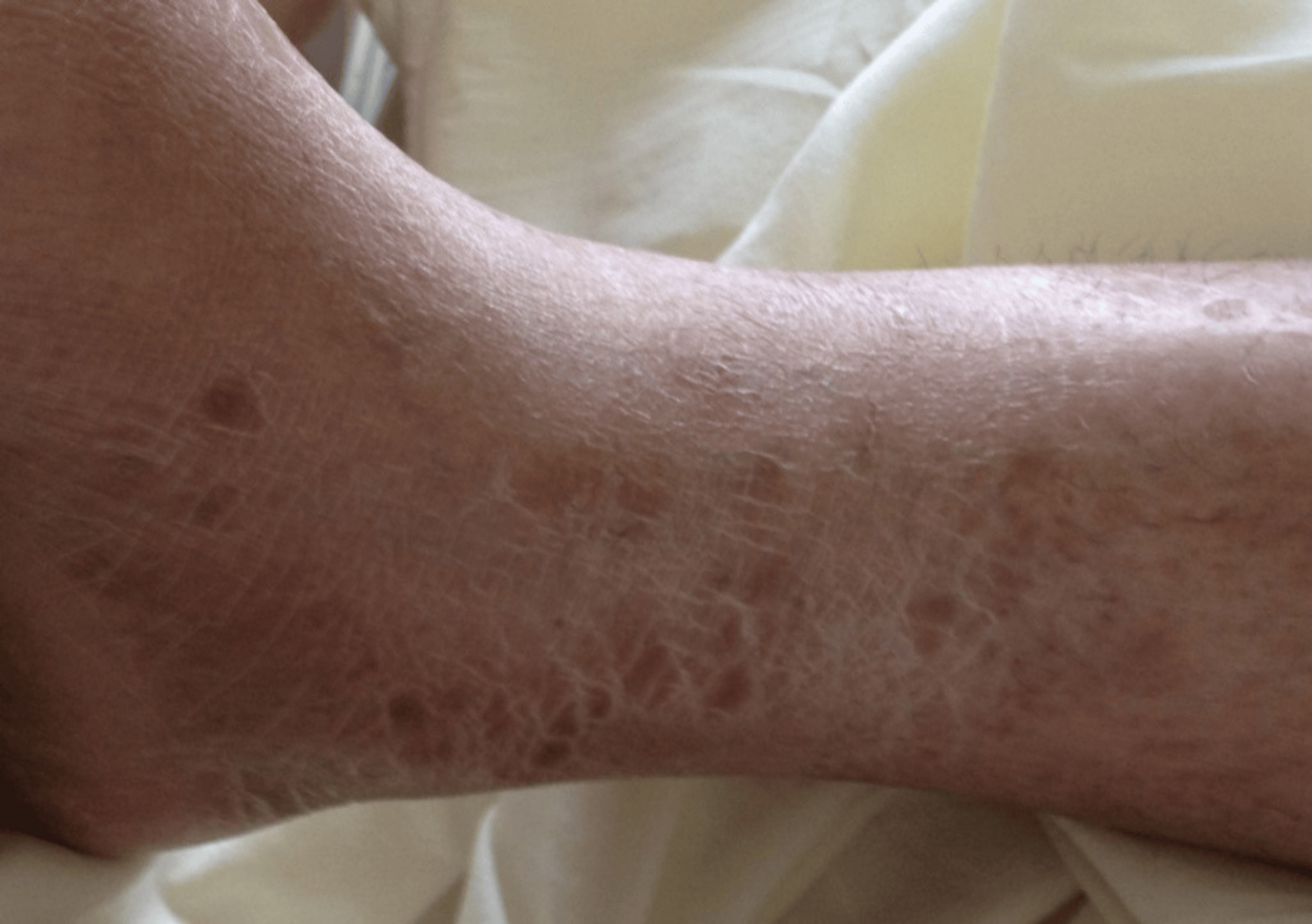
Erythematous rash over the lower limbs

A 50-year-old female presented with complaints of intermittent fever, more during evening hours, for three weeks, associated with decreased appetite and generalized weakness. Her clinical examination revealed an erythematous rash over both lower limbs (Figure 1) and scleral congestion in her eyes.

On initial examination, the patient had a heart rate of 80 beats/min, blood pressure of 120/80 mmHg measured on the right arm in the supine position, no palpable lymph nodes, and pallor was present. The cardiovascular examination was normal with no murmurs; the chest was clear, and the abdomen was soft and non-tender.

On the night of the first hospital day, she developed sudden restlessness with unrecordable blood pressure. She sustained a ventricular fibrillation arrest, requiring defibrillation and return of spontaneous circulation after 30 minutes. She was subsequently intubated, placed on mechanical ventilation, and transferred to the critical care unit for further management.

Investigation

**Figure 2:**
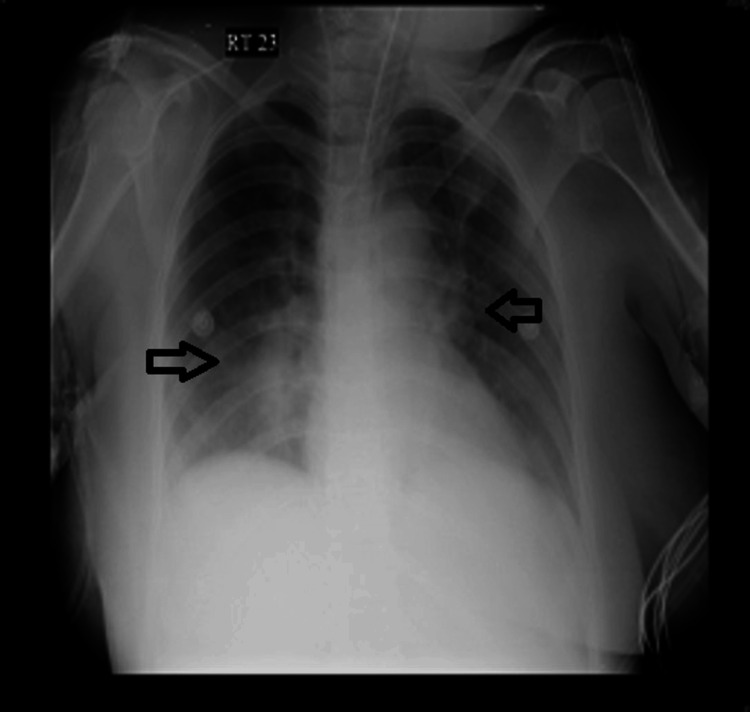
Chest radiograph done after intubation showing bilateral hilar prominence

The initial blood investigations revealed acute kidney injury (serum creatinine: 2.3 mg/dL), hyperuricemia (11.7 mg/dL), and hypercalcemia (calcium: 12.6 mg/dL; corrected calcium: 13.4 mg/dL) (Table 1). Her parathyroid hormone levels were normal, while her 25-hydroxyvitamin D levels were low. The erythrocyte sedimentation rate was elevated (56 mm/h). Liver enzymes were also elevated (serum glutamic-oxaloacetic transaminase: 76 U/L). Blood and urine cultures were sterile. Tracheal secretions showed occasional Gram-positive cocci in pairs, no acid-fast bacilli, a negative tuberculosis polymerase chain reaction test (TB-PCR), and no fungal elements on smears; cultures were sterile. The workup for tropical fever (typhoid, typhus fever, dengue, chikungunya, malaria, leptospirosis) was negative. The 24-hour Holter was normal, and echocardiography revealed no regional wall motion abnormality and a normal ejection fraction. A chest radiograph obtained after arrest showed bilateral hilar prominence (Figure 2).

**Table 1:** Laboratory investigation APTT: activated partial thromboplastin time, HCV IgG: hepatitis C virus immunoglobulin G, HIV: human immunodeficiency virus, Hbsag: hepatitis B surface antigen, ESR: erythrocyte sedimentation rate, CRP: C-reactive protein, AFB: acid-fast bacilli, M.TB PCR: *Mycobacterium tuberculosis* polymerase chain reaction, TSH: thyroid-stimulating hormone

Investigations	Values	Reference ranges
Hemoglobin (g/L)	10.8	13-17
Total leucocyte count (cell/cum)	4.5	4-10
Platelet (cell/cumm)	207	150-410
Creatinine (mg/dL)	2.3	0.9-1.3
Sodium (mmol/L)	135	136-146
Potassium (mmol/L)	3.5	3.5-5.1
Calcium (mg/dL)	12.6	8.8-10.2
Uric acid (mg/dl)	11.7	2.5-7
Aspartate aminotransferase (IU/L)	76	15-41
Alanine aminotransferase (IU/L)	31	17-63
Alkaline phosphatase (IU/L)	146	32-91
Gamma-glutamyl transferase (IU/L)	100	7-50
Globulin (g/dL)	2.5	2.3-3.5
Albumin (g/dL)	3.0	3.5-5.0
Total bilirubin (mg/dL)	1	0.1-1.2
Direct bilirubin (mg/L)	0.2	0-0.2
Indirect bilirubin (mg/dL)	0.8	0-0.8
APTT (sec)	33.8	23.7-33.5
INR (%)	1.17	sec
HCV IgG (s/co)	0.01	<0.90
HIV (I and II)	Negative	Negative
HbsAg (s/co)	0.10	<0.90
ESR (mm/hr)	56	0-15
CRP (mg/dl)	28	<3
M.TB PCR	Negative	Negative
AFB culture (tracheal secretion)	Negative	Negative
Tracehal secretion – culture and sensitivity	Negative	Negative
Tracheal secretion – fungal culture	Negative	Negative
Tracheal secretion – Gram stain	Occasional Gram-positive cocci in pairs	Negative
Procalcitonin (ng/ml)	0.3	<0.50
TSH µIU/mL	1.86	0.34-5.6

**Figure 3:**
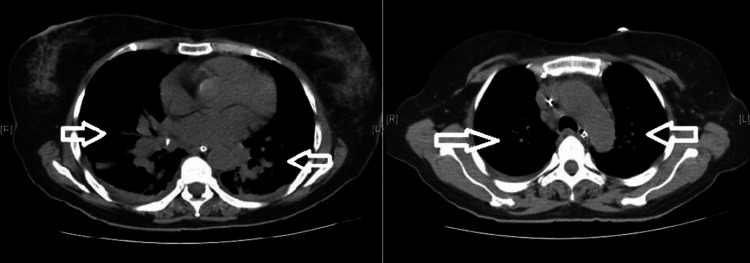
CT chest revealing enlarged lymph nodes in the pre-carinal, pre-tracheal, and subcarinal regions CT: computed tomography

A chest CT revealed enlarged lymph nodes in the pre-carinal, pre-tracheal, and subcarinal regions (Figure 3).

**Figure 4:**
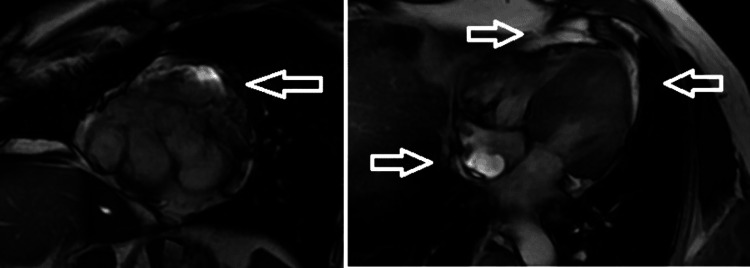
Cardiac MRI showing a delayed gadolinium enhancement suggestive of infiltrative disease MRI: magnetic resonance imaging

The angiotensin-converting enzyme (ACE) level was elevated (141.9 U/L). Serum protein electrophoresis did not show any M spike. The autoimmune workup was normal. Bilateral lower limb venous Doppler ultrasound did not reveal any evidence of deep vein thrombosis. These findings raised a suspicion of sarcoidosis. Cardiac MRI demonstrated delayed gadolinium enhancement suggestive of an infiltrative disease (Figure 4).

**Figure 5:**
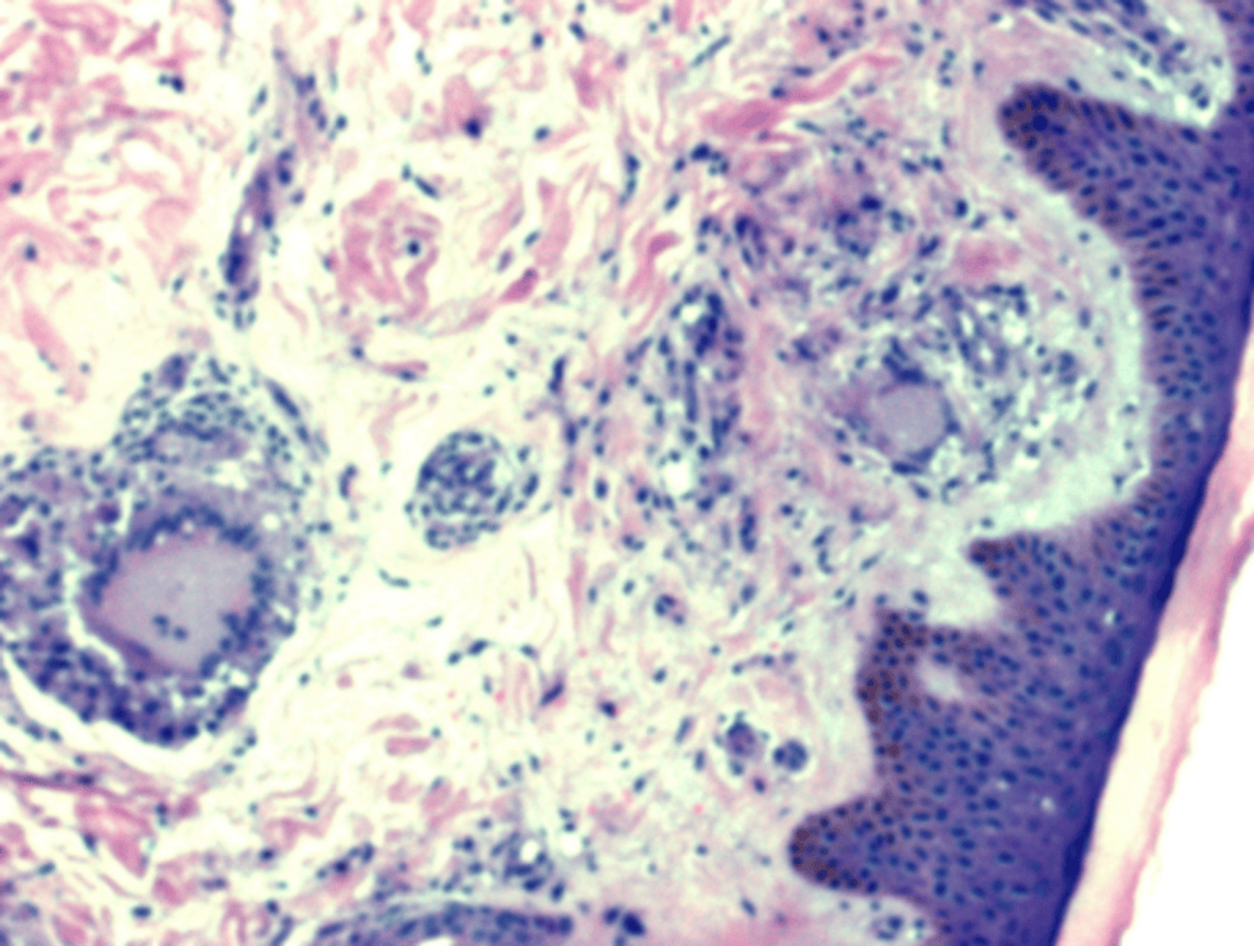
Skin biopsy taken from the site of skin lesions revealing non-caseating granulomas

A skin biopsy was taken from the site of skin lesions, which revealed non-caseating granulomas suggestive of sarcoidosis (Figure 5).

Differential diagnosis

The patient had been admitted as a case of fever under evaluation. She had been admitted for workup of the same. However, she had a cardiac arrest in the ward on the first night of her admission, which was due to ventricular fibrillation. She did not have a history of similar events in the past, and there was no family history of heart disease. The echocardiography and 24-hour Holter were normal and did not reveal any clues to the diagnosis. The workup for tropical fever (typhoid, typhus fever, dengue, chikungunya, malaria, leptospirosis) was negative. Her biochemistry showed evidence of acute kidney injury (serum creatinine: 2.3 mg/dL), hyperuricemia (11.7 mg/dL), and hypercalcemia (calcium: 12.6 mg/dL; corrected calcium: 13.4 mg/dL).

Chest radiography revealed hilar prominence. A chest CT was done to further substantiate. This showed hilar lymphadenopathy. She had an elevated ACE level. This raised the possibility of sarcoidosis and tuberculosis. The tracheal secretion was sent for microscopy/staining and TB-PCR. The microscopy showed no acid-fast bacilli, and TB-PCR was negative. This made the diagnosis of tuberculosis less likely, especially in the context of sudden-onset ventricular fibrillation (cardiac involvement) with hypercalcemia, elevated ACE levels, hilar lymphadenopathy, and a skin rash with vague constitutional symptoms. We decided to do a cardiac MRI, which showed delayed gadolinium enhancement suggestive of infiltrative disease. We did a serum electrophoresis, which showed no M spikes to rule out the unlikely possibility of multiple myeloma. The most likely possibility was sarcoidosis with cardiac involvement. We decided to perform a skin biopsy (a minimally invasive option) from the rash on the leg. This showed a non-caseating granuloma. This helped in establishing a diagnosis of systemic sarcoidosis with cardiac involvement.

Treatment

She was initially started on intravenous antibiotics (ceftriaxone 2 g twice daily) as per local sensitivity guidelines, due to an initial suspicion of tropical fevers. After the diagnosis of sarcoidosis was established, she was started on intravenous systemic steroids (methylprednisolone 40 mg once daily), and intravenous antibiotics were discontinued. The patient was extubated 48 hours after the arrest. She was subsequently transitioned to oral prednisolone at a dose of 0.5 mg/kg/day.

She was advised to undergo intraventricular conduction device (IVCD) insertion, as recommended by the Heart Rhythm Society Expert Consensus Statement on the Diagnosis and Management of Arrhythmias Associated With Cardiac Sarcoidosis [1]. Patients with arrest due to ventricular fibrillation should be considered for IVCD insertion. Due to financial constraints, she was referred to a government center for the procedure and was maintained on oral amiodarone until IVCD insertion could be performed.

Follow-up

She was followed up for a period of six weeks. Her acute kidney injury, hypercalcemia, and hyperuricemia had resolved, and the skin lesions and scleritis had improved. She was clinically well during follow-up. Steroid tapering was initiated after four weeks of treatment because of the remarkable response and resolution of symptoms, and she was continued on a maintenance dose of steroids. Azathioprine was added as a steroid-sparing agent. She is scheduled for IVCD insertion at the government center. An FDG-PET scan was advised at three months; however, she has not been able to undergo the procedure because of financial limitations.

## Discussion

Sarcoidosis is a multisystem granulomatous disorder characterized by the formation of non-caseating granulomas in various organs, most commonly the lungs, lymph nodes, eyes, skin, and heart. CS is an infiltrative cardiomyopathy caused by granulomatous inflammation of the myocardium [1]. CS is reported in up to 25% of patients with systemic sarcoidosis [1]. The heart is the third most commonly affected organ after the lungs and lymph nodes. The exact prevalence of sarcoidosis in India is not well established; however, previous studies have reported an annual case load of 10-12 per 1,000 registrations in Delhi and 61.2 per 100,000 new registrations in Kolkata [2]. In the United States, the annual incidence of systemic sarcoidosis is 10.9 per 100,000 among the White population and 35.5 per 100,000 among the African-American population, with cardiac involvement reported in 5-7% of these patients [1]. Isolated CS refers to cardiac involvement in the absence of systemic sarcoidosis [1].

The pathophysiology of sarcoidosis involves activation of macrophages and CD4+ T lymphocytes, leading to increased production of tumor necrosis factor-alpha (TNF-α), interleukin-2 (IL-2), interferon-gamma (IFN-γ), and serum amyloid A protein [1]. IL-2 promotes the expansion of activated lymphocytes, while TNF-α and IFN-γ stimulate macrophage accumulation and activation. Activated macrophages subsequently release additional immunomodulatory molecules, including TNF-α, IL-1, IL-6, IL-8, IL-12, IL-15, and IL-18, which drive granuloma formation and chronic inflammation [3].

Granulomas in CS are found predominantly in the interventricular septum and inferior left ventricle. They may involve the epicardial, myocardial, and, less commonly, endocardial layers, with the subepicardium and myocardium being the most frequent sites of involvement. Endocardial involvement is rare. Granulomas within the interventricular septum can disrupt the conduction system, resulting in atrioventricular block, bundle branch block, ventricular arrhythmias, and supraventricular arrhythmias, particularly atrial fibrillation [4]. Sarcoidosis involving the left ventricle may lead to dilated cardiomyopathy, mitral valve dysfunction, papillary muscle involvement, and left ventricular aneurysms.

CS carries a poor prognosis, with left ventricular ejection fraction (LVEF) being the most important prognostic indicator [1]. Advanced imaging modalities such as cardiac MRI and FDG-PET form the mainstay of diagnosis.

Echocardiography may reveal thinning of the basal septum, akinetic or dyskinetic regional wall motion abnormalities, left ventricular systolic dysfunction, left ventricular dilation, and aneurysm formation. However, its utility is limited by operator dependence and low sensitivity for detecting early disease. Echocardiographic abnormalities have been reported in 14-46% of patients with sarcoidosis [5].

Cardiac MRI is an important diagnostic modality in CS. Typical findings include wall thinning, aneurysm formation, and chamber dilation. Late gadolinium enhancement (LGE) is a particularly valuable feature, as it detects myocardial fibrosis and serves as a poor prognostic indicator. The reported sensitivity of cardiac MRI ranges from 75% to 100%, with a specificity of 76-100% [1].

Cardiac MRI is highly sensitive for detecting both myocardial edema (on T2-weighted imaging) and scarring (on LGE), providing detailed structural and tissue characterization. In contrast, FDG-PET is more sensitive for detecting active inflammation, especially in the early stages of disease, and can demonstrate inflammatory activity even in the absence of structural abnormalities on cardiac MRI.

A meta-analysis by Hulten et al. demonstrated the prognostic significance of LGE. The annualized incidence of cardiovascular mortality was 1.9% in LGE-positive patients compared with 0.3% in LGE-negative patients. The annualized incidence of ventricular arrhythmia was 5.9% versus 0%, and overall mortality was 8.8% versus 0.6% in LGE-positive versus LGE-negative patients, respectively [6].

FDG-PET is another important investigative tool in CS. It is typically performed in a staged manner, with both cardiac and whole-body imaging, which assists in identifying biopsy sites with active inflammation. FDG uptake may appear focal, diffuse, or patchy, with or without associated perfusion abnormalities, depending on the presence of active inflammation or scar formation. The reported sensitivity of FDG-PET is approximately 89%, with a specificity of 78% [1]. However, several pitfalls exist, including the non-specific nature of FDG uptake, difficulty in distinguishing physiologic from pathologic uptake, and challenges in patient preparation and image interpretation. Furthermore, other cardiac conditions, such as myocarditis or myocardial infarction, may also demonstrate FDG uptake, making differentiation from sarcoidosis challenging.

Endomyocardial biopsy demonstrating non-caseating granulomas remains the diagnostic gold standard. However, the yield is low (25-36%) because of the patchy and focal nature of the disease [1].

The mainstay of treatment is corticosteroid therapy. Oral prednisone is typically initiated at 0.5-1 mg/kg daily (maximum dose: 60 mg/day). Tapering is guided by clinical response, with a common approach being a reduction of 10 mg per month to reach a target of 20 mg daily by the time of the first follow-up FDG-PET scan, usually at approximately three months. Steroid-sparing agents are considered in cases of significant corticosteroid toxicity, when high doses are required for disease control or when corticosteroids alone prove inadequate. These agents help minimize long-term steroid-related side effects. Commonly used steroid-sparing agents include azathioprine, cyclophosphamide, and leflunomide. Biologic immunotherapies such as infliximab, adalimumab, and rituximab are reserved as third- or fourth-line options because of their potential complications [1].

Pacemaker implantation should be considered in patients with CS who develop reversible conduction block. An implantable cardioverter-defibrillator (ICD) is recommended for patients with CS who have experienced spontaneous sustained ventricular arrhythmias, including prior cardiac arrest, or for those with a LVEF <35% despite optimal medical therapy and immunosuppression [1]. IVCD insertion is recommended in accordance with the Heart Rhythm Society Expert Consensus Statement on the Diagnosis and Management of Arrhythmias Associated With Cardiac Sarcoidosis. Patients with ventricular fibrillation arrest should be considered for IVCD insertion [1].

We also reviewed several previously published case reports that provide important clinical insights into the diagnosis and management of CS. These are summarized in Table 2.

**Table 2:** Summary of interesting case reports CMR: cardiac magnetic resonance imaging, FDG-PET: fluorodeoxyglucose positron emission tomography, ICD: implantable cardioverter-defibrillator, ECG: electrocardiogram, VT: ventricular tachycardia, CS: cardiac sarcoidosis, LGE: late gadolinium enhancement

Case report	Details	Learning points
Riasat et al. (2022) [7]	A female patient presented with syncope and experienced multiple episodes of cardiac arrest during her hospital stay. Laboratory tests, including an autoimmune workup, were negative. CMR and FDG-PET revealed intramyocardial delayed enhancement of the basal anteroseptal region and patchy areas of increased uptake in the anteroseptal and inferior myocardial segments. The patient was treated with methylprednisolone, and a follow-up FDG-PET scan demonstrated resolution of myocardial uptake.	CMR and FDG-PET are key diagnostic modalities for CS.
Godo et al. (2022) [8]	A 68-year-old man presented to the hospital with cardiac arrest secondary to ventricular fibrillation. He was successfully resuscitated. Coronary angiography revealed normal coronary arteries, while intracoronary acetylcholine provocation testing demonstrated both epicardial and microvascular coronary spasm. An FDG-PET scan showed increased myocardial fluorodeoxyglucose uptake, and CMR revealed LGE. Based on these findings, he was diagnosed with isolated CS. The patient was treated with a calcium-channel blocker for coronary artery spasm and prednisolone for CS, and an ICD was inserted.	A high index of suspicion is essential for diagnosing CS.
Rosenfeld et al. (2021) [9]	A 57-year-old man presented with palpitations and syncope. His ECG demonstrated a prolonged PR interval of 370 ms. Echocardiography revealed moderate right ventricular dilation, a left ventricular ejection fraction of 45-50%, and anteroseptal hypokinesis. Coronary angiography was normal. An electrophysiological study identified monomorphic VT originating from the septum. FDG-PET showed focal uptake in the septum and right ventricle. Endomyocardial biopsy confirmed the diagnosis of CS. The patient underwent implantation of an ICD and was started on corticosteroid therapy. A follow-up FDG-PET scan demonstrated resolution of abnormal uptake. Despite this, he continued to experience symptomatic VT of multiple morphologies, which was refractory to multiple antiarrhythmic drugs and immunosuppression. Extensive ablation was performed, but proved unsuccessful. The patient ultimately died due to complications of VT storm.	This case highlights the complexity of diagnosing CS, and despite optimal treatment, the prognosis can remain poor.

## Conclusions

A multidisciplinary team approach is essential in the management of CS due to the complex nature of the disease and its potential multi-organ involvement. Early diagnosis improves prognosis, but this requires a high index of suspicion. Peripheral signs of sarcoidosis, such as skin manifestations (erythema nodosum, lupus pernio), uveitis, and peripheral arthritis, should not be overlooked, as these may serve as early diagnostic clues, particularly when present alongside cardiac symptoms. This case highlights the diagnostic challenges of CS and underscores the risk of sudden cardiac death. Clinicians should remain vigilant, especially when evaluating patients with febrile illness and vague symptoms without a clear etiology.

Cardiac MRI and FDG-PET are the most important noninvasive modalities for diagnosing CS. FDG-PET also helps identify viable biopsy sites, providing a less invasive alternative to endomyocardial biopsy, and can detect sites of active inflammation in the heart to guide diagnosis, treatment decisions, and monitoring of therapeutic response. Management focuses on reducing inflammation, controlling symptoms, and preventing complications. Corticosteroids remain the mainstay of therapy, as they can reduce inflammation and may prevent or reverse ventricular dysfunction. Device therapy (such as pacemakers or defibrillators) may be required in patients with ventricular arrhythmias or advanced heart block. Regular follow-up and monitoring are crucial to identify and manage complications effectively.
